# Induction of Bim and Bid gene expression during accelerated apoptosis in severe sepsis

**DOI:** 10.1186/cc7088

**Published:** 2008-10-16

**Authors:** Stefan U Weber, Jens-Christian Schewe, Lutz E Lehmann, Stefan Müller, Malte Book, Sven Klaschik, Andreas Hoeft, Frank Stüber

**Affiliations:** 1 Department of Anesthesiology and Intensive Care Medicine, University Bonn Medical Center, Sigmund-Freud-Straße 25, 53129 Bonn, Germany; 2 Center for Biologics Evaluation and Research (CBER), Food and Drug Administration, 1401 Rockville Pike, Suite 200N, Rockville, MD 20852-1448, USA; 3 Department of Anaesthesiology and Pain Therapy, University Hospital Bern "Inselspital", 3010 Bern, Switzerland

## Abstract

**Introduction:**

In transgenic animal models of sepsis, members of the Bcl-2 family of proteins regulate lymphocyte apoptosis and survival of sepsis. This study investigates the gene regulation of pro-apoptotic and anti-apoptotic members of the Bcl-2 family of proteins in patients with early stage severe sepsis.

**Methods:**

In this prospective case-control study, patients were recruited from three intensive care units (ICUs) in a university hospital. Sixteen patients were enrolled when they fulfilled the criteria of severe sepsis. Ten critically ill but non-septic patients and 11 healthy volunteers served as controls. Blood samples were immediately obtained at inclusion. To confirm the presence of accelerated apoptosis in the patient groups, caspase-3 activation and phosphatidylserine externalisation in CD4+, CD8+ and CD19+ lymphocyte subsets were assessed using flow cytometry. Specific mRNAs of Bcl-2 family members were quantified from whole blood by real-time PCR. To test for statistical significance, Kruskal-Wallis testing with Dunn's multiple comparison test for post hoc analysis was performed.

**Results:**

In all lymphocyte populations caspase-3 (p < 0.05) was activated, which was reflected in an increased phosphatidylserine externalisation (p < 0.05). Accordingly, lymphocyte counts were decreased in early severe sepsis. In CD4+ T-cells (p < 0.05) and B-cells (p < 0.001) the Bcl-2 protein was decreased in severe sepsis. Gene expression of the BH3-only Bim was massively upregulated as compared with critically ill patients (p < 0.001) and 51.6-fold as compared with healthy controls (p < 0.05). Bid was increased 12.9-fold compared with critically ill patients (p < 0.001). In the group of mitochondrial apoptosis inducers, Bak was upregulated 5.6-fold, while the expression of Bax showed no significant variations. By contrast, the pro-survival members Bcl-2 and Bcl-xl were both downregulated in severe sepsis (p < 0.001 and p < 0.05, respectively).

**Conclusions:**

In early severe sepsis a gene expression pattern with induction of the pro-apoptotic Bcl-2 family members Bim, Bid and Bak and a downregulation of the anti-apoptotic Bcl-2 and Bcl-xl proteins was observed in peripheral blood. This constellation may affect cellular susceptibility to apoptosis and complex immune dysfunction in sepsis.

## Introduction

There has been accumulating evidence both in animals and humans, that apoptosis of lymphocytes [1,2], monocytes [3], dendritic cells [4] and gut epithelial cells [5] is accelerated in severe sepsis and contributes to temporary immunosuppression [6]. Sepsis is a systemic disorder driven by a dysregulation of the immune system, that clearly displays pro-inflammatory as well as anti-inflammatory signatures [7]. The temporary immunosuppression during sepsis is still considered an unsolved problem in the treatment of sepsis because during this phase, patients often succumb to secondary infections, caused by pathogens that are not necessarily virulent in healthy individuals [6]. In multiple animal models of sepsis, inhibition of lymphocyte apoptosis has been shown to dramatically improve survival [5,8-10].

Apoptosis is a type of cell death that follows a tightly regulated program. It results in the disposal of the apoptotic cell without spilling toxic intracellular components and is the central mechanism for tissue homeostasis during development [11]. The cell death program may be activated via a membrane bound pathway, where the signal is initiated by so called 'death receptors' [12] or via the mitochondrion [13]. Pro-apoptotic compounds are usually sequestered in the intermembrane space [14]. On permeabilisation of the outer mitochondrial membrane, these proteins are released into the cytosol forming the apoptosome and subsequently activating caspase-9. Ultimately, both pathways converge in the activation of caspase-3, the caspase that is the main executioner, [15] which has multiple targets and degrades structural and functional proteins [11].

The integrity of the mitochondrial membrane is controlled by members of the Bcl-2 protein family [16]. Bcl-2 is the prototype of a large family of related proteins displaying pro-apoptotic or anti-apoptotic properties. The related proteins are divided into three groups: anti-apoptotic Bcl-2-like proteins such as Bcl-2 and Bcl-xl, which share four regions of homology, the BH1-4 domains; pro-apoptotic Bax-like proteins sharing only domains 1–3 [17]; and the group of BH3-only proteins, which contain only domain 3 and function as sensors and transducers of apoptotic signals (eg, Bim and Bid) [16]. The proteins Bak and Bax form oligomers that perturb the outer mitochondrial membrane. To keep them inactive they are bound by pro-survival guards [18]. According to the displacement model, binding of the mobile BH3-only protein Bim to pro-survival members of the Bcl-2 family liberates Bak/Bax from their guards, thereby rendering them active [16].

Several lines of evidence point to the involvement of the mitochondrion and the Bcl-family of proteins in the induction of lymphocyte apoptosis during sepsis. Overexpression of Bcl-2 or adoptive transfer of Bcl-2 overexpressing lymphocytes in murine models reduces mortality from caecal ligation puncture-induced sepsis [19]. In patients, Bcl-2 expression was found to be decreased during sepsis [1,20,21]. Recently, it was shown in animal models, that BH3-only proteins are involved in the induction of apoptosis during sepsis: Bim -/- and Bid -/- mice exhibited both reduced apoptosis and improved survival in murine sepsis as compared with respective wild-type animals [22].

Taking into account the importance of Bcl-2 family members for the transduction of apoptosis signals in animal models, we hypothesised that diverse members of the Bcl-2 family underlie transcriptional regulation in humans during sepsis. Therefore, we studied the gene expression of important members of Bcl-2-like, Bax-like and BH3-only proteins of the Bcl-2 family during a phase of accelerated lymphocyte apoptosis in human patients experiencing severe sepsis. The presence of accelerated apoptosis in the study collective was confirmed by phosphatidylserine externalisation and caspase-3 activation in lymphocyte subpopulations.

## Materials and methods

### Patients and controls

This study was conducted with approval from the ethics board of the University of Bonn, Germany. Patients were included after written informed consent was given by them or their next-of-kin. The study included 16 patients who had been treated in three surgical intensive care units (ICUs) at the university hospital of Bonn after a diagnosis of severe sepsis according to the American College of Chest Physicians/Society of Critical Care Medicine consensus criteria [23]. A blood sample was obtained from each patient on the day of inclusion. Ten patients treated in the ICU without sepsis or signs of infection served as controls. For both groups exclusion criteria were a lack of informed consent, age under 18 years, and preexisting haematological or immunological disease. The basic clinical characteristics of the critically ill patients and patients with severe sepsis (Table 1) have been published previously in part [24]. As a second control group, 11 healthy subjects (six males, five females, median age = 34 years) were tested. All patients were Caucasian.

**Table 1:** Clinical data of patient groups

	Critically ill non-septic n = 10	Severe sepsis n = 16	p
Age	61 +/- 5	56 +/- 4	ns

APACHE II score at inclusion	12 +/- 1	26 +/- 2	< 0.05

SAPS II score at inclusion	23 +/- 2	57 +/- 4	< 0.01

SOFA score at inclusion	3 +/- 1	15 +/- 1	< 0.001

Mechanically ventilated at inclusion (n)	6	16	

Vasopressor treatment at inclusion (n)	0	13	

Antibiotic treatment at inclusion (n)	8	16	

Steroid dose* 3 mg/kg/24hours (n)	0	8	

Serum interleukin-6 at inclusion (pg/ml)	20.77 +/- 13.37	4304 +/- 2466	< 0.001

Serum procalcitonin at inclusion (μg/l)	0.25 +/- 0.18	39.38 +/- 23.56	< 0.001

White blood cell count at inclusion (G/l)	11.94 +/- 1.17	14.94 +/- 3.09	ns

Lymphocyte count at inclusion (G/l]	1.47 +/- 0.19	0.88 +/- 0.24	< 0.05

Mean +/- standard error of the mean. APACHE II = Acute Physiology and Chronic Health Evaluation II; ns = not significant; SAPS II = Simplified Acute Physiology Score II; SOFA = Sepsis related Organ Failure Assessment.

* hydrocortisone; before or at the time of sample acquisition.

### Sample acquisition and storage

Blood samples were obtained within four hours after severe sepsis was first diagnosed to sample early stage severe sepsis. For flow cytometry, samples were anticoagulated with ethylenediaminetetraacetic acid (EDTA) and processed within 30 minutes. For RNA quantification, 2.5 ml whole blood was collected using blood RNA tubes (Paxgene system, PreAnalytiX, Qiagen, Hilden, Germany) and stored at -80°C until extraction. This method stabilised the RNA until further analysis.

### Phosphatidylserine externalisation

Phosphatidylserine externalisation was measured by annexin V binding. Samples of 100 μl fresh whole blood were subjected to lysis with an ammonium chloride solution (Becton Dickinson, Heidelberg, Germany) for 15 minutes at room temperature to substantially lyse red blood cells without affecting the leucocyte populations. After washing, cells were phenotyped with phycoerythrin-labelled antibodies directed against CD4, CD8 and CD19 (Becton Dickinson, Heidelberg, Germany). They were then incubated with annexin-V-fluorescein-isothiocyanate (Becton Dickinson, Heidelberg, Germany) for 10 minutes at room temperature in Hepes buffer containing calcium (2.5 mM). Before flow cytometric analysis, cells were labelled with the DNA-dye 7-actinoaminomycin (7-AAD; Becton Dickinson, Heidelberg, Germany) for the detection of membrane leaks and subjected to data acquisition within 10 minutes. Cells that were phosphatidylserine positive but 7-AAD negative were selected by gating.

### Caspase-3 activation and Bcl-2 expression

For caspase-3 activation and Bcl-2 expression measurement, samples of 100 μl whole blood were analysed. Erythrocytes were lysed with 1.5 ml PharmLyse, an ammonium chloride-based lysing reagent. (Pharmingen, Heidelberg, Germany) at room temperature. All subsequent steps were carried out at 4°C. Cells were washed with PBS (Sigma-Aldrich, Steinheim, Germany) and fixed with 750 μl PBS containing 4% paraformaldehyde (Sigma-Aldrich, Steinheim, Germany). After fixation, cells were permeabilised with 1ml PBS containing 0.5% saponin (Sigma-Aldrich, Steinheim, Germany) and 1% BSA (Sigma-Aldrich, Steinheim, Germany). Cells were washed twice with PBS (0.5% saponin/1% BSA). Active caspase-3 was detected by a phycoerythrin-labelled antibody directed against the active fragment (Becton Dickinson, Heidelberg, Germany). Bcl-2 was detected with a specific phycoerythrin-conjugated monoclonal antibody (Becton Dickinson, Heidelberg, Germany). Isotype control antibodies (Becton Dickinson, Heidelberg, Germany) were used to determine unspecific binding. After washing cells were phenotyped antibodies against CD4, CD8 and CD19 that were labelled with peridinin-chlorophyll-protein (PerCP; Becton Dickinson, Heidelberg, Germany). Cells were washed twice and resuspended in 250 μl PBS (0.5% saponin/1% BSA) for flow cytometric analysis.

### Flow cytometry

Stained and fixed cells were measured by flow cytometry. Data were acquired in a flow cytometer (FACSCalibur, Becton Dickinson, Heidelberg, Germany) and analysed via CellQuest Pro software (CellQuest Pro, Version 4.0.2, Becton Dickinson, Heidelberg, Germany). Ten-thousand cells of interest were acquired. Populations of interest were selected by multiple gating using forward (cell size) and sideward (cell granularity) scatter and green or red regions on fluorescence channels. Antibody-binding was expressed as mean fluorescence intensity corrected for nonspecific binding using matched isotype control antibodies. Results of caspase-3 staining were given as percentages of positive cells. In case of Bcl-2 staining, daily calibration curves were generated using latex beads coupled to defined quantities of phycoerythrin (Quantibrite, Becton Dickinson, Heidelberg, Germany). Fluorescence intensities were calculated accordingly to compensate for potential deviations from linearity of the cytometer and to reduce day to day variability. Since the Bcl-2 antibody used, like most conjugated antibodies, is not guaranteed to be coupled to phycoerythrin in a one-to-one ratio, results were not given as molecules per cell but as linear fluorescence units.

### RNA isolation and cDNA preparation

Total RNA was extracted from whole blood using a PAXgene Blood RNA kit (Qiagen, Hilden, Germany) according to the manufacturer's instructions. cDNA was synthesised with a 1^st ^Strand cDNA Synthesis Kit for RT-PCR (Roche Diagnostics, Mannheim, Germany). The reaction mixture contained 8.2 μl of total RNA (equivalent to about 500 ng), 5 mM magnesium chloride, 2.1 mM deoxyribonucleotide triphosphate (dNTP), 3.2 μg of random primer p(dN)_6 _(0.04 A_260 _units/μl), 6.50 units of RNase inhibitor, 20 units of avian myeloblastosis virus reverse transcriptase, and one sample of reaction buffer to a total volume of 20 μl. The reaction was incubated as follows: 25°C for 10 minutes, 42°C for 60 minutes, 99°C for five minutes and 4°C for five minutes.

### Real-time PCR

Real-time PCR with cDNA from blood samples was performed according to the manufactuer's manual in a total volume of 20 μl on a LightCycler instruments using the Light-Cycler FastStart DNA^Plus ^SYBR Green I (both from Roche Diagnostics, Mannheim, Germany). For each sample, reactions were performed in duplicates for target genes. Fluorescence was monitored at the end of the second segment of each cycle. PCR reactions were performed under the following conditions: the reactions started with an initial denaturation at 95°C for 10 minutes followed by 45 amplification cycles; Bcl-2: 95°C for 15 seconds, 65°C for five seconds, 72°C for five seconds followed by an additional heating to 85°C for melting curve detection.

The conditions for Bcl-xl and Bax differed only in annealing temperatures of 64°C and 70°C, respectively. Bid and Bak PCR reactions were performed at 95°C for 10 seconds, 60°C for 30 seconds and 72°C for one second. Bim PCR reaction was performed at 95°C for 15 seconds, 58°C for 30 seconds and 72°C for one second. Then, a melting curve was created for each reaction, and the product was cooled at 40°C for 30 seconds.

To calculate the amounts of transcripts relative to the housekeeping gene h-HPRT, housekeeping gene PCR was performed using the Light-Cycler-h-HPRT Housekeeping Gene Set (Roche Diagnostics, Mannheim, Germany) according to the manufacturer's instructions. For relative quantification analyses the expression target mRNA in each sample was calculated relative to the housekeeping gene using the LightCycler quantification software (Roche Diagnostics, Mannheim, Germany). An external calibrator was added in duplicates to each run to compensate for inter-run variability. PCR products were cloned into Jm109 plasmids (Promega, Mannheim, Germany) according to the manufacturer's instructions.

For specific genes the following primers were used: HPRT 5'-TGACCTTGATTTATTTTGCATACC, 5'-CGAGCAAGACGTTCAGTCCT (Operon, Cologne, Germany); Bim Refseq No. NM_006538 Band Size: 123 bp Reference Positions: 15–35 (SuperArray, Frederick, MD, USA), Bid Refseq No. NM_001196 Band Size: 176 bp Reference Positions: 331–351 (SuperArray, Frederick, MD, USA), Bak Refseq No. NM_001188, Band Size: 176 bp Reference Positions: 405–424 (SuperArray, Frederick, MD, USA), Bcl-2 5'-GCC AGC TGC ACC TGA CGC CCT TC, 5'-CCG CAT GCT GGG GCC GTA CAG TT (271 bp), Bcl-xl 5'-CAC AGT CAT GCC CGT CAG G, 5'-TGA ATG AAC TCT TCC GGG ATG (281 bp), Bax 5'-ACC CGG TGC CTC AGG ATG CGT, 5'-ACC CGG TGC CTC AGG ATG CGT (185 bp).

Dilution curves of PCR quantifications of cloned PCR products were created for initial calibration of the software. For quantification the crossing point method was employed. Concentrations were calculated as "normalised ratio" with Relative Quantification Software (Roche Diagnostics, Mannheim, Germany).

$$normalised.ratio=\frac{conc.t\arg et(sample)}{conc.reference(sample)}:\frac{conc.t\arg et(calibrator)}{conc.reference(calibrator)}$$

The mean expression level of each gene was set at the value 100 for healthy controls and relative changes in critically ill and sepsis patients were calculated accordingly.

### Statistics

To detect differences between clinical characteristics of the two patient groups a Mann-Whitney test was employed. To evaluate differences between the three groups, healthy controls, non-septic critically ill patients and patients experiencing severe sepsis, a non-Gaussian-distribution was assumed. Consequently, Kruskal-Wallis testing was performed. For post-hoc testing Dunn's multiple comparison test was employed when appropriate. Correlations were tested using the Spearman procedure and a two-tailed p value. Values were given as mean +/- standard error of the mean if not indicated. p < 0.05 was considered statistically significant.

## Results

### Patients and clinical parameters

Sixteen patients with severe sepsis (11 males and 5 females) and 10 critically ill non-septic patients (eight males and two females) were enrolled. The basic clinical characteristics of the critically ill patients and patients with severe sepsis are listed in Table 1 and have been published previously in part [24]. Overall, 8 of 16 patients in the sepsis group died during the ICU stay. Patients with severe sepsis and non-critically ill patients did not differ in age but they did differ in Acute Physiology and Chronic Health Evaluation (APACHE) II score, simplified acute physiology score (SAPS) II and Sequential Organ Failure Assessment (SOFA) score.

Furthermore, patients with severe sepsis had higher interleukin (IL) 6 and higher procalcitonin serum concentrations. Briefly, underlying diseases of the sepsis group were necrotising fasciitis (n = 2), faecal peritonitis (n = 8) and pneumonia (n = 6). Nine of the ten critically ill non-septic patients were included in their post-operative period after trauma, abdominal or pharyngeal cancer, or aortic aneurysm rupture with a delayed recovery. These patients received prophylactic antibiotic treatment with no signs of infection in the perioperative phase. One patient had abacterial pancreatitis and did not receive antibiotics. Patients did not receive immunosuppressants or drotrecogin alfa (activated) before or during their treatment. Eight patients with severe sepsis received 3 mg/kg hydrocortisone before or at the time of sampling. No difference in white blood cells counts was found comparing critically ill patients with patients with sepsis. However, lymphocyte counts were decreased in severe sepsis as compared with critically ill patients and dropped below the local reference range of 1–4 G/l (Table 1).

### Phosphatidylserine externalisation and caspase-3 activation

**Figure 1:**
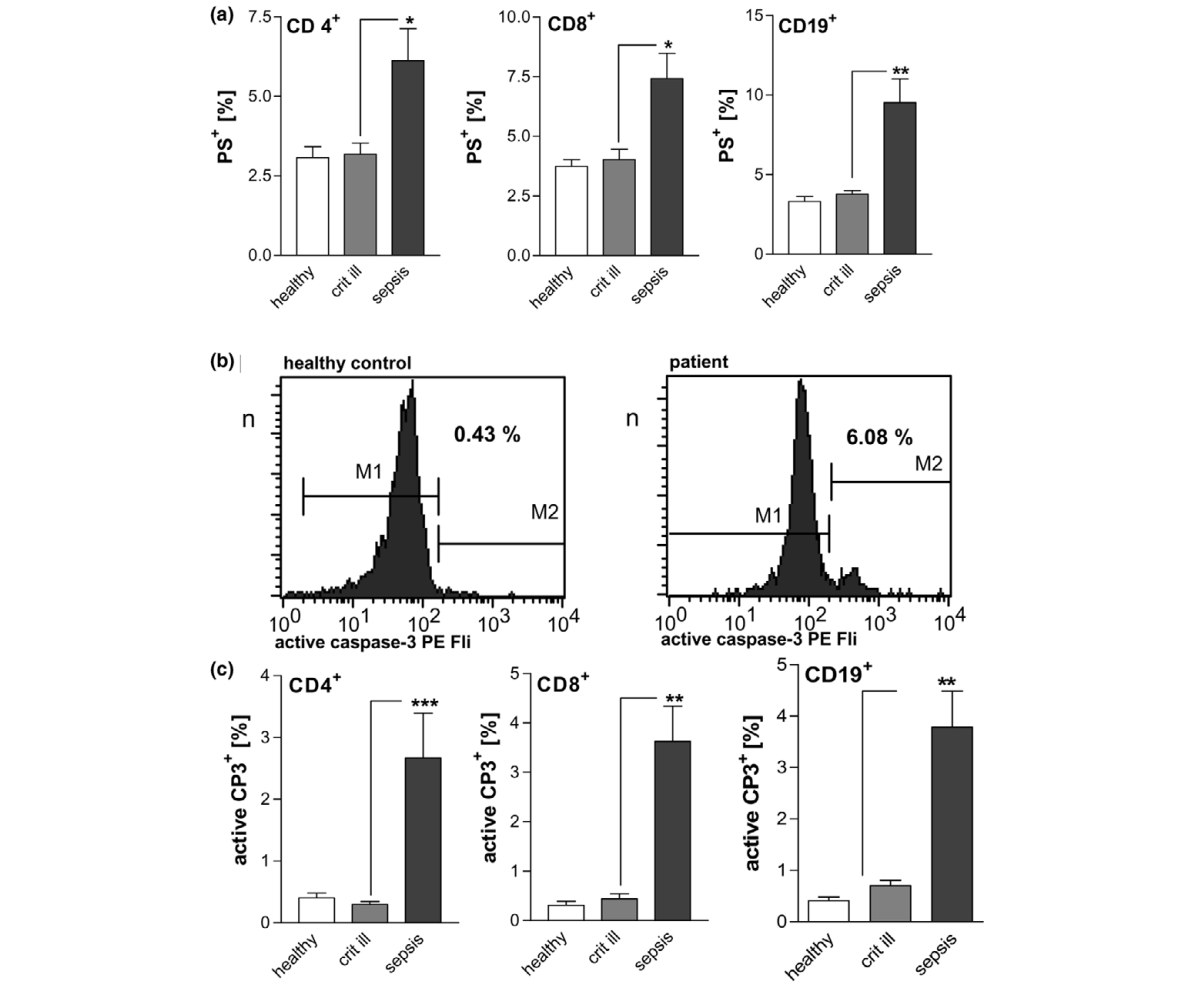
**Confirmation of accelerated apoptosis in patients with severe sepsis**. (a) Phosphatidylserine externalisation on CD4^+^, CD8^+ ^and CD19^+ ^lymphocytes of patients with severe sepsis. The population of phosphatidylserine-positive lymphocytes was increased as compared with critically ill patients and healthy controls. *p < 0.05, **p < 0.01. (b) Representative histograms of caspase-3 activation. Intracellular active caspase-3 was stained with a phycoerythrin-conjugated monoclonal antibody. CD 4^+ ^T-cells were selected via immunophenotyping in flow cytometry. Region M2 describes the population with activated caspase-3. (c) Detection of active caspase-3 in lymphocyte populations. In patients with severe sepsis the phosphatidylserine-positive lymphocytes (CD 4^+ ^T-cells, CD 8^+ ^T-cells, CD19^+ ^B-cells) were increased as compared with critically ill patients and healthy controls. **p < 0.01, ***p < 0.001.

Phosphatidylserine externalisation marks cells for phagocytosis as an early event of the apoptotic process. Cells were considered early apoptotic when phosphatidylserine was externalised on cells with a still intact membrane, as indicated by negative staining for 7-AAD. CD4^+ ^and CD8^+ ^T-cells and CD19^+ ^B-cells exhibited significantly raised portions of phosphatidylserine-positive populations in severe sepsis, but not in critically ill patients (Figure 1a).

Caspase-3 is the central executioner caspase. Activation of caspase-3 leads to degradation of multiple intracellular substrates and to the typical morphological features of classical apoptosis. In the current study, activation of caspase-3 was measured by an antibody specific to the active fragment of cleaved caspase-3 (Figures 1b and 1c). In patients with severe sepsis, the subpopulation with active caspase-3 was elevated in CD4+ T-cells and CD8+ T-cells compared with critically ill patients or healthy controls. Also, B-cells from septic patients were found to contain significantly more activated caspase-3 than B-cells from critically ill patients or healthy controls.

### Bcl-2 expression

**Figure 2:**
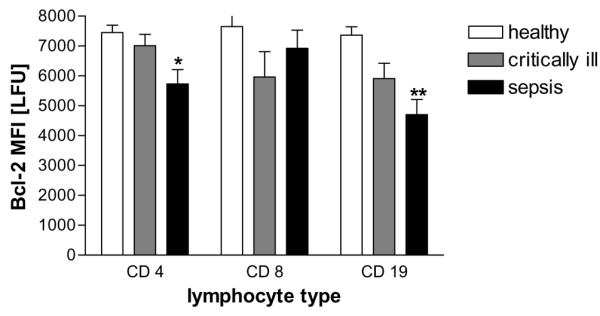
**Expression of Bcl-2 in severe sepsis**. The expression of the Bcl-2 protein was measured by flow cytometry in CD4^+^, CD8^+ ^and CD19^+ ^lymphocytes. In CD4^+ ^and CD 19^+ ^lymphocytes, a significant reduction in Bcl-2 was observed in severe sepsis. *p < 0.05, **p < 0.01

Since the Bcl-2 family of proteins is known to regulate the mitochondrial integrity, we analysed the expression of Bcl-2 (Figure 2). The amount of Bcl-2 in CD4^+ ^T-cells of critically ill non-septic patients did not differ significantly from healthy controls. However, in patients with sepsis, Bcl-2 protein levels dropped by about 25%. In CD8^+ ^T-cells, no significant change between the three groups could be observed. The decrease in mitochondrial Bcl-2 was most pronounced in B-cells, where Bcl-2 dropped by 36% when compared with healthy controls.

### mRNA expression of Bcl-2 family members

**Figure 3:**
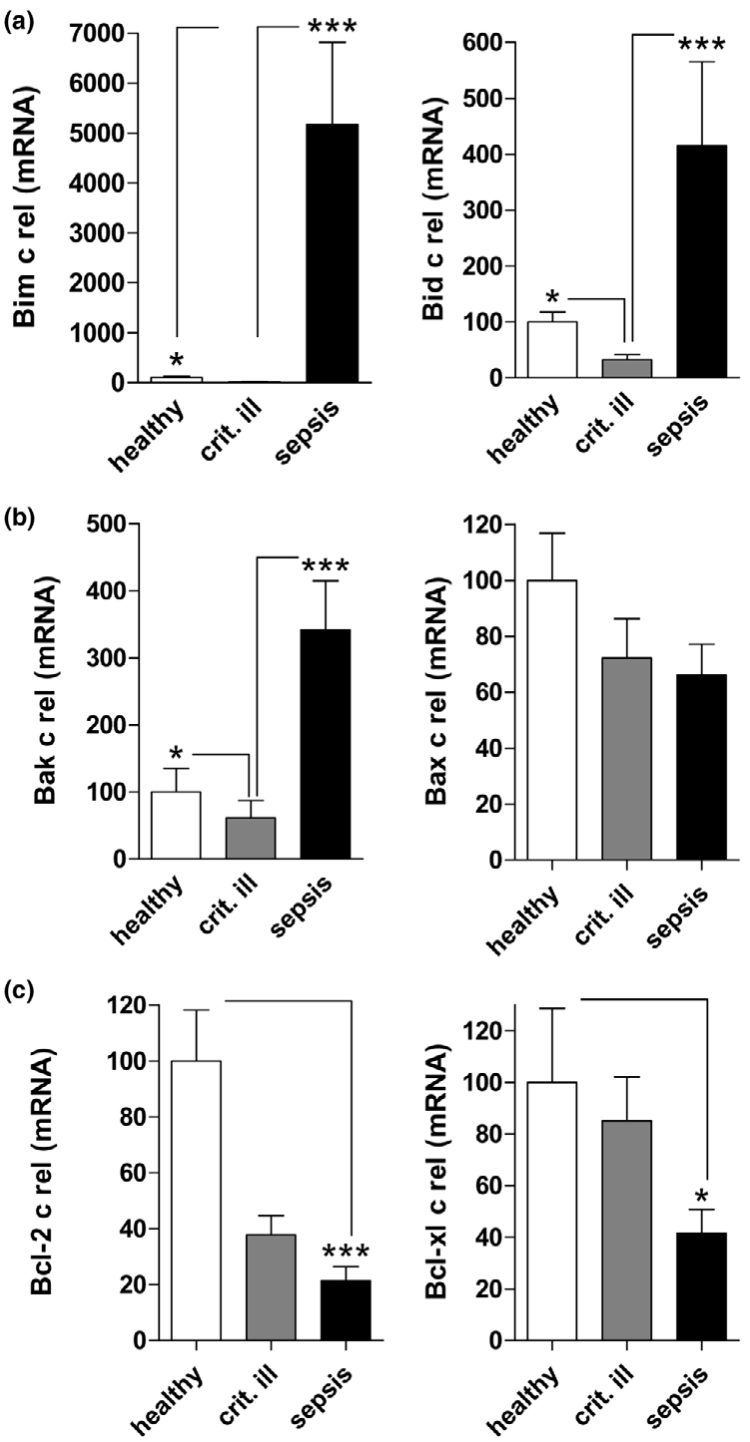
**mRNA expression of Bcl-2 family members**. mRNA expression was measured by real-time PCR in patients with severe sepsis, critically ill patients and healthy controls. (a) The expression of BH3-only members Bim and Bid was upregulated, (b) the pro death-group Bak and Bax was regulated heterogeneously and (c) the anti-apoptotic Bcl-2 and Bcl-xl were downregulated. *p < 0.05, *p < 0.001

When investigating the mRNA expression of mobile pro-apoptotic BH3-only proteins of the Bcl-2 family, massive induction was observed in severe sepsis (Figure 3a). When compared with healthy controls and critically ill patients, mRNA expression of Bim was upregulated. This corresponds to a 310.5-fold increase compared with critically ill patients and a 51.7-fold rise compared with healthy controls. While Bid was decreased in critically ill patients, it was markedly upregulated in severe sepsis.

Analysing pro-apoptotic members of the Bcl-2 family located in the mitochondrial membrane (Figure 3b), a lower expression of Bak was observed in critically ill patients, while a highly significant elevation of Bak mRNA was found in sepsis patients. Bax mRNA expression was not changed significantly in severe sepsis as compared with control patients.

The expression of pro-survival molecules was reduced in severe sepsis (Figure 3c). The mRNA expression of Bcl-2 was markedly downregulated in patients with severe sepsis as compared with healthy controls. Bcl-2 expression in critically ill patients was found at an intermediate level, which showed no statistical significance. The mRNA of the anti-apoptotic Bcl-xl decreased in severe sepsis as compared with critically ill patients.

When comparing survivors versus non-survivors no differences were observed for Bim (survivors 5548 ± 2510 versus non-survivors 4784 ± 2307, p > 0.5), Bid (538.3 ± 228.2 versus 291.8 ± 97.11, p > 0.5), and Bak (409.1 ± 124.6 versus 273.7 ± 79.17, p > 0.5). Corticoid treatment had no significant effect on gene expression of Bim (corticoid treatment 5493 ± 2417 versus no corticoid treatment 4839 ± 2406, p > 0.5), Bid (267.3 ± 102.2 versus 562.8 ± 283.1, p > 0.5) or Bak (343.9 ± 123.4 versus 338.8 ± 88.79, p = 1).

The degree of organ failure, as expressed by the SOFA score, correlated with pro-apoptotic Bim, Bid and Bax gene expression (Table 2). The severity of illness (SAPS II score) correlated with Bim and Bak gene expression (Table 2). Furthermore, mRNA expression of Bim, Bid and Bak demonstrated a positive correlation with procalcitonin, although Bim and Bid correlated with serum IL-6 (Table 2).

**Table 2:** Correlation of pro-apoptotic gene expression with clinical parameters.

	**Bim**		**Bid**		**Bak**	
	
	**r**_s_	**p**	**r**_s_	**p**	**r**_s_	**p**
**SAPS II**	0.49	<0.05		ns	0.40	<0.05

**SOFA**	0.533	<0.01	0.40	<0.05	0.44	<0.05

**Interleukin-6**	0.57	<0.01	0.43	<0.05		ns

**Procalcitonin**	0.56	<0.01	0.44	<0.05	0.41	<0.05

ns = not significant; p = two-tailed p value; r_s _= Spearman correlation coefficient; SAPS II = Simplified Acute Physiology Score II; SOFA = Sepsis related Organ Failure Assessment.

## Discussion

The current study demonstrates that pro-apoptotic and anti-apoptotic members of the Bcl-2 family are regulated differentially in a cohort of sepsis patients exhibiting accelerated lymphocyte apoptosis as compared with critically ill non-septic patients. The patients with sepsis in the study exhibited accelerated apoptosis of all major lymphocyte subpopulations. The degree of apoptosis as indicated by phosphatidylserine externalisation and caspase-3 activation was comparable with findings of previous reports [1,25,26]. Thus the sepsis cohort of this study exhibited a degree of apoptosis typical of severe sepsis.

Besides death receptor mediated mechanisms [9,27,28], mitochondrial events play a crucial role in the initiation of leucocyte apoptosis during sepsis [1] and their possible involvement in the apoptotic process during sepsis was the focus of this study. On the side of the pro-death Bcl-2 family, Bim and Bid were analysed because they are mobile and are sufficient to activate the mitochondrial apoptosis pathway. For the group of membrane-associated pro-death members, Bax and Bak were analysed. As anti-apoptotic members, Bcl-2 and Bcl-xl gene expression was measured. A typical pattern of gene expression of the Bcl-2 family was observed in patients with severe sepsis as compared with the two control groups: on the one hand the BH-3-only pro-death gene expression of Bim and Bid was massively upregulated. Furthermore, the mRNA of the pro-apoptotic Bak was increased. On the other hand, the genes of the protective Bcl-2 and Bcl-xl were expressed at lower levels.

The BH3-only Bim is the principal regulator of haematopoietic homeostasis [17] and a potent inducer of death [29]. Bim can bind promiscuously to Bcl-2 pro-survival proteins, so it has a dominant role in apoptosis induction in response to diverse cytotoxic stimuli [16]. Release of Bim from the dynein motor complex is regarded as an apoptosis initiating event, because it does not require caspase activity [30]. Absence of Bim in Bim -/- mice almost prevented apoptosis after sepsis induction and improved survival markedly [22]. Also, siRNA-mediated inhibition of Bim gene expression decreased lymphocyte apoptosis and improved survival in a mouse model of sepsis [31].

In the current study, Bim was upregulated massively in patients with severe sepsis. This event could be a possible initiator of the accelerated apoptosis observed in the study. On the transcriptional level, C-Jun N-terminal kinase and the forkhead transcription factor FKHR-L1 are known to regulate Bim expression in other models [32]. Our study is limited by the fact that we did not analyse the activity of relevant transcription factors and post-transcriptional regulation. These questions need to be examined in a follow-up study.

Bid transfers apoptosis signals from the extrinsic to the mitochondrial pathway of apoptosis [33]. On cleavage by caspase-8, truncated Bid translocates to the mitochondrion [34]. From mouse experiments it is known that lymphocyte apoptosis in Bid -/- animals after caecal ligation puncture-induced sepsis is reduced slightly [22]. Still, survival of sepsis in these animals is improved significantly [22]. Therefore, it is possible that the marked upregulation of Bid in our patients with severe sepsis contributed to a worse outcome.

Bak and Bax belong to the main pro-death members of the Bcl-2 group. Although Bax, in accordance with Bilbault and colleagues [20], was not regulated, Bak mRNA expression was markedly increased in our patients with severe sepsis. Apoptosis initiates when BH3-only ligands, such as Bim, engage multiple Bcl-2 homologues, which liberate Bak to execute mitochondrial membrane opening [35]. Bim and Bak are known to synergise in the induction of apoptosis of haematopoietic cells [36].

In our study we confirmed the downregulation of Bcl-2 in severe sepsis. Moreover, we also found Bxl-xl mRNA to be reduced. The lack of Bxl-xl is likely to be of clinical significance, because treatment with TAT-Bcl-xl, a cell permeable construct, improved the survival of mice in the model using caecal ligation puncture-induced sepsis [37]. Interestingly, also IL-6 plays a role in the expression of Bcl-2 and Bcl-xl, because critical levels of liver Bcl-2 and Bcl-xl could not be maintained in IL-6 deficient mice. The sepsis patients in this study had high concentrations of IL-6. Still, in our patients IL-6 could not maintain the levels of Bcl-2 and Bcl-xl. Another mechanism for the downregulation of Bcl-2 and Bcl-xl in sepsis has been described recently by Groesdonk and colleagues [38]. It was shown that the DNA-binding activity of nuclear factor kB was reduced in T-cells of septic mice; however, sufficient expression of these anti-apoptotic proteins is dependent on the canonical activation of nuclear factor kB.

Although mRNA was extracted from whole blood, possible contributions to the gene expression pattern by neutrophils cannot be ruled out. In sepsis, however, apoptosis is delayed [39] and is associated with the maintenance of mitochondrial transmembrane potential [40] suggesting an anti-apoptotic pattern of gene expression of the Bcl-2 family. Accordingly, in a rat model of sepsis Bim on neutrophils was down-regulated and Bcl-xl was increased [41]. The opposite was observed in our study. Thus, it is probable that the massive upregulation of pro-apoptotic gene expression is generated by mononuclear cells rather than neutrophils.

In principle, glucocorticoids may induce apoptosis of T-cells, especially in immunocompromised patients with HIV infection [42]. On the other hand glucocorticoids may rescue CD4^+ ^lymphocytes from activation-induced apoptosis triggered by HIV [43]. In our study, no influence of low-dose corticoid treatment on apoptotic gene expression was found. This is in line with findings by Hotchkiss and colleagues, who observed no effect of low-dose glucocorticoid treatment on rates of lymphocyte apoptosis in severe sepsis [1].

Gene expression of Bim, and in part of the other pro-apoptotic Bcl-2 family members, correlated with the severity of disease, the degree of organ failure and the pro-inflammatory response (IL-6). It also correlated with serum procalcitonin, which is regarded as a marker of infection rather than inflammation. Our study design did not compare septic and non-septic patients of the same degree of critical illness. Therefore, it cannot be ruled out that severely ill patients with high SAPS II and SOFA scores but no indication of infection may also exhibit accelerated lymphocyte apoptosis and pro-apoptotic gene regulation. The phenomenon of accelerated apoptosis is not unique to sepsis but is also found in other types of critical illness. For example, burn injuries preferentially induce CD4^+ ^T-cell apoptosis [44] and haemorrhagic shock induces splenocyte apoptosis [45]. Further studies should also investigate Bcl-2 family expression patterns under these conditions.

Besides mitochondrial events involving the Bcl-2 family several other potential inducers of lymphocyte apoptosis are discussed in sepsis [22,34]. In regular lymphocyte homeostasis, life and death balance is maintained by activation-induced cell death, which is induced via death receptors of the tumour necrosis factor receptor family or T-cell receptor activation [46]. The death receptor Fas is upregulated [47] on leucocytes in sepsis patients and caspase-8 is active [22] in severe sepsis in animals. Inhibition of Fas or silencing of caspase-8 inhibits apoptosis during sepsis [9]. Interestingly, cross-talk of Fas-receptor signalling via truncated Bid may contribute to apoptosis induction via the mitochondrion [34]. Although activation-induced cell death is a probable candidate to explain apoptosis in sepsis, in murine experimental sepsis it was recently shown that accelerated lymphocyte apoptosis does not require activation and T-cell receptor engagement [48]. Another member of the tumour necrosis factor receptor family, CD40 is expressed on B- and T-lymphocytes during sepsis [49]. Inhibition of CD40 by an antagonising antibody inhibits lymphocyte apoptosis and improves survival of murine experimental sepsis. Interestingly, at least in part, this protection is attributed to increased expression of the anti-apoptotic Bcl-2 family member Bcl-xl [49].

## Conclusion

A pro-apoptotic pattern of gene expression in the Bcl-2 family was observed in severe sepsis. The combination of an increase of Bim, Bid and Bad in conjunction with lowered levels of Bcl-2 and Bcl-xl may render cells more susceptible to apoptosis during sepsis. This study underlines the potential importance of the Bcl-2 family and mitochondrial events in the induction and transduction of apoptosis during sepsis. BH3-only proteins such as Bim and Bid may be relevant therapeutic targets in future strategies to inhibit accelerated apoptosis in patients with severe sepsis.

## Key messages

• Gene expression of the Bcl-2 family during accelerated apoptosis in severe sepsis displays a pro-apoptotic pattern compared with critically ill non-septic patients and healthy controls.

• Expression of pro-apoptotic Bim, Bid and Bak mRNA is markedly upregulated.

• Expression of anti-apoptotic Bcl-xl and Bcl-2 is downregulated.

## Abbreviations

7-AAD: 7-actinoaminomycin; APACHE II:Acute Physiology and Chronic Health Evaluation II; bp: base pair; BSA: boveine serum albumin; dNTP: deoxyribonucleotide triphosphate; EDTA: ethylenediaminetetraacetic acid; ICU: intensive care unit; IL: interleukin; PBS: phosphate buffered saline; PCR: polymerase chain reaction; SAPS II: Simplified Acute Physiology Score II; SOFA: Sepsis related Organ Failure Assessment.

## Competing interests

The authors declare that they have no competing interests.

## Authors' contributions

SW and JS conceived the study, participated in its design, established the flow cytometric methodology, participated in data acquisition, participated in the data analysis and drafted the manuscript. LL and MB established the PCR methodology and designed the primers. SM and SK carried out flow cytometry analysis. AH and FS participated in the study design and data analysis. LL, MB, SM, SK, AH and FS critically revised the manuscript for intellectual content. All authors read and approved the final manuscript.

## References

[B1] Hotchkiss RS, Osmon SB, Chang KC, Wagner TH, Coopersmith CM, Karl IE (2005). Accelerated lymphocyte death in sepsis occurs by both the death receptor and mitochondrial pathways. J Immunol.

[B2] Schroeder S, Lindemann C, Decker D, Klaschik S, Hering R, Putensen C, Hoeft A, von Ruecker A, Stuber F (2001). Increased susceptibility to apoptosis in circulating lymphocytes of critically ill patients. Langenbecks Arch Surg.

[B3] Adrie C, Bachelet M, Vayssier-Taussat M, Russo-Marie F, Bouchaert I, Adib-Conquy M, Cavaillon JM, Pinsky MR, Dhainaut JF, Polla BS (2001). Mitochondrial membrane potential and apoptosis peripheral blood monocytes in severe human sepsis. Am J Respir Crit Care Med.

[B4] Efron PA, Martins A, Minnich D, Tinsley K, Ungaro R, Bahjat FR, Hotchkiss R, Clare-Salzler M, Moldawer LL (2004). Characterization of the systemic loss of dendritic cells in murine lymph nodes during polymicrobial sepsis. J Immunol.

[B5] Coopersmith CM, Stromberg PE, Dunne WM, Davis CG, Amiot DM, Buchman TG, Karl IE, Hotchkiss RS (2002). Inhibition of intestinal epithelial apoptosis and survival in a murine model of pneumonia-induced sepsis. JAMA.

[B6] Hotchkiss RS, Nicholson DW (2006). Apoptosis and caspases regulate death and inflammation in sepsis. Nat Rev Immunol.

[B7] Hotchkiss RS, Karl IE (2003). The pathophysiology and treatment of sepsis. N Engl J Med.

[B8] Hotchkiss RS, Chang KC, Swanson PE, Tinsley KW, Hui JJ, Klender P, Xanthoudakis S, Roy S, Black C, Grimm E, Aspiotis R, Han Y, Nicholson DW, Karl IE (2000). Caspase inhibitors improve survival in sepsis: a critical role of the lymphocyte. Nat Immunol.

[B9] Wesche-Soldato DE, Chung CS, Lomas-Neira J, Doughty LA, Gregory SH, Ayala A (2005). In vivo delivery of caspase-8 or Fas siRNA improves the survival of septic mice. Blood.

[B10] Oberholzer C, Oberholzer A, Bahjat FR, Minter RM, Tannahill CL, Abouhamze A, LaFace D, Hutchins B, Clare-Salzler MJ, Moldawer LL (2001). Targeted adenovirus-induced expression of IL-10 decreases thymic apoptosis and improves survival in murine sepsis. Proc Natl Acad Sci USA.

[B11] Hengartner MO (2000). The biochemistry of apoptosis. Nature.

[B12] Krammer PH (2000). CD95's deadly mission in the immune system. Nature.

[B13] Zamzami N, Kroemer G (2001). The mitochondrion in apoptosis: how Pandora's box opens. Nat Rev Mol Cell Biol.

[B14] Harris MH, Thompson CB (2000). The role of the Bcl-2 family in the regulation of outer mitochondrial membrane permeability. Cell Death Differ.

[B15] Thornberry NA, Lazebnik Y (1998). Caspases: enemies within. Science.

[B16] Willis SN, Adams JM (2005). Life in the balance: how BH3-only proteins induce apoptosis. Curr Opin Cell Biol.

[B17] Cory S, Adams JM (2002). The Bcl2 family: regulators of the cellular life-or-death switch. Nat Rev Cancer.

[B18] Willis SN, Chen L, Dewson G, Wei A, Naik E, Fletcher JI, Adams JM, Huang DC (2005). Proapoptotic Bak is sequestered by Mcl-1 and Bcl-xL, but not Bcl-2, until displaced by BH3-only proteins. Genes Dev.

[B19] Hotchkiss RS, Tinsley KW, Swanson PE, Chang KC, Cobb JP, Buchman TG, Korsmeyer SJ, Karl IE (1999). Prevention of lymphocyte cell death in sepsis improves survival in mice. Proc Natl Acad Sci USA.

[B20] Bilbault P, Lavaux T, Lahlou A, Uring-Lambert B, Gaub MP, Ratomponirina C, Meyer N, Oudet P, Schneider F (2004). Transient Bcl-2 gene down-expression in circulating mononuclear cells of severe sepsis patients who died despite appropriate intensive care. Intensive Care Med.

[B21] Bilbault P, Lavaux T, Launoy A, Gaub MP, Meyer N, Oudet P, Pottecher T, Jaeger A, Schneider F (2007). Influence of drotrecogin alpha (activated) infusion on the variation of Bax/Bcl-2 and Bax/Bcl-xl ratios in circulating mononuclear cells: a cohort study in septic shock patients. Crit Care Med.

[B22] Chang KC, Unsinger J, Davis CG, Schwulst SJ, Muenzer JT, Strasser A, Hotchkiss RS (2007). Multiple triggers of cell death in sepsis: death receptor and mitochondrial-mediated apoptosis. Faseb J.

[B23] Bone RC, Balk RA, Cerra FB, Dellinger RP, Fein AM, Knaus WA, Schein RM, Sibbald WJ (1992). Definitions for sepsis and organ failure and guidelines for the use of innovative therapies in sepsis. The ACCP/SCCM Consensus Conference Committee. American College of Chest Physicians/Society of Critical Care Medicine. Chest.

[B24] Book M, Chen Q, Lehmann LE, Klaschik S, Weber S, Schewe JC, Luepertz M, Hoeft A, Stuber F (2007). Inducibility of the endogenous antibiotic peptide beta-defensin 2 is impaired in patients with severe sepsis. Crit Care.

[B25] Le Tulzo Y, Pangault C, Gacouin A, Guilloux V, Tribut O, Amiot L, Tattevin P, Thomas R, Fauchet R, Drenou B (2002). Early circulating lymphocyte apoptosis in human septic shock is associated with poor outcome. Shock.

[B26] Roth G, Moser B, Krenn C, Brunner M, Haisjackl M, Almer G, Gerlitz S, Wolner E, Boltz-Nitulescu G, Ankersmit HJ (2003). Susceptibility to programmed cell death in T-lymphocytes from septic patients: a mechanism for lymphopenia and Th2 predominance. Biochem Biophys Res Commun.

[B27] Wesche DE, Lomas-Neira JL, Perl M, Chung CS, Ayala A (2005). Leukocyte apoptosis and its significance in sepsis and shock. J Leukoc Biol.

[B28] Ayala A, Xin XY, Ayala CA, Sonefeld DE, Karr SM, Evans TA, Chaudry IH (1998). Increased mucosal B-lymphocyte apoptosis during polymicrobial sepsis is a Fas ligand but not an endotoxin-mediated process. Blood.

[B29] Bouillet P, Metcalf D, Huang DC, Tarlinton DM, Kay TW, Kontgen F, Adams JM, Strasser A (1999). Proapoptotic Bcl-2 relative Bim required for certain apoptotic responses, leukocyte homeostasis, and to preclude autoimmunity. Science.

[B30] Puthalakath H, Huang DC, O'Reilly LA, King SM, Strasser A (1999). The proapoptotic activity of the Bcl-2 family member Bim is regulated by interaction with the dynein motor complex. Mol Cell.

[B31] Schwulst SJ, Muenzer JT, Peck-Palmer OM, Chang KC, Davis CG, McDonough JS, Osborne DF, Walton AH, Unsinger J, McDunn JE, Hotchkiss RS (2008). BIM siRNA decreases lymphocyte apoptosis and improves survival in sepsis. Shock.

[B32] Puthalakath H, Strasser A (2002). Keeping killers on a tight leash: transcriptional and post-translational control of the pro-apoptotic activity of BH3-only proteins. Cell Death Differ.

[B33] Gross A (2006). BID as a double agent in cell life and death. Cell Cycle.

[B34] Perl M, Chung CS, Ayala A (2005). Apoptosis. Crit Care Med.

[B35] Willis SN, Fletcher JI, Kaufmann T, van Delft MF, Chen L, Czabotar PE, Ierino H, Lee EF, Fairlie WD, Bouillet P, Strasser A, Kluck RM, Adams JM, Huang DC (2007). Apoptosis initiated when BH3 ligands engage multiple Bcl-2 homologs, not Bax or Bak. Science.

[B36] Hutcheson J, Scatizzi JC, Bickel E, Brown NJ, Bouillet P, Strasser A, Perlman H (2005). Combined loss of proapoptotic genes Bak or Bax with Bim synergizes to cause defects in hematopoiesis and in thymocyte apoptosis. J Exp Med.

[B37] Hotchkiss RS, McConnell KW, Bullok K, Davis CG, Chang KC, Schwulst SJ, Dunne JC, Dietz GP, Bahr M, McDunn JE, Karl IE, Wagner TH, Cobb JP, Coopersmith CM, Piwnica-Worms D (2006). TAT-BH4 and TAT-Bcl-xL peptides protect against sepsis-induced lymphocyte apoptosis in vivo. J Immunol.

[B38] Groesdonk HV, Wagner F, Hoffarth B, Georgieff M, Senftleben U (2007). Enhancement of NF-kappaB activation in lymphocytes prevents T cell apoptosis and improves survival in murine sepsis. J Immunol.

[B39] Fialkow L, Fochesatto Filho L, Bozzetti MC, Milani AR, Rodrigues Filho EM, Ladniuk RM, Pierozan P, de Moura RM, Prolla JC, Vachon E, Downey GP (2006). Neutrophil apoptosis: a marker of disease severity in sepsis and sepsis-induced acute respiratory distress syndrome. Crit Care.

[B40] Taneja R, Parodo J, Jia SH, Kapus A, Rotstein OD, Marshall JC (2004). Delayed neutrophil apoptosis in sepsis is associated with maintenance of mitochondrial transmembrane potential and reduced caspase-9 activity. Crit Care Med.

[B41] Guo RF, Sun L, Gao H, Shi KX, Rittirsch D, Sarma VJ, Zetoune FS, Ward PA (2006). In vivo regulation of neutrophil apoptosis by C5a during sepsis. J Leukoc Biol.

[B42] Roger PM, Perbost I, Ticchioni M, Fuzibet JG, Breittmayer JP, Durant J, Pesce A, Bernard A, Dellamonica P (2004). Apoptosis of naive CD4+ T-cells from HIV-infected patients with poor immune response to HAART is enhanced in vitro by steroid. J Infect.

[B43] Lu W, Salerno-Goncalves R, Yuan J, Sylvie D, Han DS, Andrieu JM (1995). Glucocorticoids rescue CD4+ T lymphocytes from activation-induced apoptosis triggered by HIV-1: implications for pathogenesis and therapy. Aids.

[B44] Patenaude J, D'Elia M, Hamelin C, Garrel D, Bernier J (2005). Burn injury induces a change in T cell homeostasis affecting preferentially CD4+ T cells. J Leukoc Biol.

[B45] Hostmann A, Jasse K, Schulze-Tanzil G, Robinson Y, Oberholzer A, Ertel W, Tschoeke SK (2008). Biphasic onset of splenic apoptosis following hemorrhagic shock: critical implications for Bax, Bcl-2, and Mcl-1 proteins. Crit Care.

[B46] Krammer PH, Arnold R, Lavrik IN (2007). Life and death in peripheral T cells. Nat Rev Immunol.

[B47] Papathanassoglou ED, Moynihan JA, McDermott MP, Ackerman MH (2001). Expression of Fas (CD95) and Fas ligand on peripheral blood mononuclear cells in critical illness and association with multiorgan dysfunction severity and survival. Crit Care Med.

[B48] Unsinger J, Herndon JM, Davis CG, Muenzer JT, Hotchkiss RS, Ferguson TA (2006). The role of TCR engagement and activation-induced cell death in sepsis-induced T cell apoptosis. J Immunol.

[B49] Schwulst SJ, Grayson MH, DiPasco PJ, Davis CG, Brahmbhatt TS, Ferguson TA, Hotchkiss RS (2006). Agonistic monoclonal antibody against CD40 receptor decreases lymphocyte apoptosis and improves survival in sepsis. J Immunol.

